# New Trends in Vascular Imaging

**DOI:** 10.3390/diagnostics11010112

**Published:** 2021-01-12

**Authors:** Kristoffer Lindskov Hansen, Jonathan Frederik Carlsen

**Affiliations:** 1 Department of Diagnostic Radiology, Copenhagen University Hospital, Rigshospitalet, 2100 Copenhagen, Denmark; jonathan.frederik.carlsen@regionh.dk; 2 Department of Clinical Medicine, University of Copenhagen, 2100 Copenhagen, Denmark

## 1. Introduction

Blood flow is essential to life and intertwined with all processes in the human body. Therefore, pathologic processes taking place in any organ will be reflected in altered flow and vasculature. From lack of flow in ischemia, increased flow and new vessels in tumors and inflammation, flow outside the vessels in extravasation, to reduced flow in stenoses. Vascularization and flow are measured and looked upon in many ways and evaluated with various methods. 

The imaging of the vasculature is an old discipline going back to the fifties, with the introduction of angiography using iodine injected into the bloodstream and visualized with an X-ray. Later came methods like ultrasound, CT, and MRI for more advanced vascular imaging techniques. The focus on flow visualization and assessment has increased with the development of more refined imaging techniques, which this special edition reflects. With new vascular imaging methods, new insight into pathology can be gained, faster and more precise diagnostics upheld, and better outcomes for patients are expected. 

The vascular research field is vast and diverse, and attempting to refine various fundamental imaging parameters, e.g., spatial and temporal resolution, conspicuity, signal-to-noise ratio, and field-of-view. The vascular system can be evaluated by qualitative and quantitative measures, but vascular imaging methods are shifting towards quantification. Apart from improving conventional flow measures like velocity and volume flow, new measures are investigated such as flow complexity and vorticity, perfusion, and vessel density and tortuosity.

In this special edition both case series, original research, and reviews are presented. The topics cover various vascular geometries using ultrasound, CT, MRI, and angiography [1,2,3,4,5,6,7,8,9,10]. 

## 2. Ultrasound

Vascular imaging with ultrasound is of high interest. Exams with ultrasound are non-invasive, rapid, and dynamic. For several decades, new methods of angle independent velocity estimation with ultrasound called Vector Flow Imaging (VFI) have been explored in the attempt to expand the conventional Doppler ultrasound. Preliminary studies have indicated that VFI is more accurate, precise, and easier to operate than conventional spectral Doppler ultrasound. With VFI, grading of stenosis can be done by assessing flow complexity as shown for the ascending aorta, the carotid and femoral artery [4]. The angle independent velocity estimation opens up for real-time quantification of vortices found anywhere in the cardiovascular system, and preliminary VFI studies have concerned evaluations of vortex formation in the heart of newborns and adults, and in vessels of straight and complex geometries to understand and map the complex fluid dynamic. The main message of these studies is that flow is far more complex and dynamic than previously described with the conventional Doppler, and that common measures such as velocity and volume flow only describe one dimension of the blood flow.

A new branch of vascular imaging, using contrast enhanced ultrasound, is called Super Resolution Imaging (SRI) and can visualize the microvasculature. The method was initially used for brain scans, coupling brain perfusion to brain activity, and stroke outcome to treatment response. Others have used the method to map the microvasculature in rodent kidneys before and after ischemic events [1]. The major goal of SRI is to capture small changes in prefusion and could be used in the assessment of, for example, diabetes and cancer.

## 3. Computed Tomography (CT)

A novel CT technique for vascular imaging is based on data acquisition from two different energy levels. As opposed to conventional CT where only a single energy level is used for imaging, Dual Energy CT (DECT) uses the attenuation measurements acquired at different energy spectra to differentiate and quantify material composition with the use of known changes in attenuation between the two spectra. In DECT, iodine can be separated and, with reconstruction algorithms, quantified as the iodine concentration, which may be used for assessment of tissue perfusion, tumor differentiation, and treatment response [9].

Reconstructions of virtual non-contrast are possible with iodine detection in DECT. Hence, extravasation will be easier to evaluate in both cranial and thoraco-abdominal scans with reduced X-ray exposure as non-contrast series can be omitted. Of particular interest is the study of acute aortic syndrome, where extravasation, intramural hematoma, and endo-leak can be challenging to image accurately with conventional CT. 

Perfusion of the lung tissue is also covered by DECT using iodine detection. Chronic obstructive pulmonary disease and pulmonary embolism can be assessed by measuring defects in lung perfusion, i.e., the direct hemodynamic effect of a pulmonary embolus is evaluated by inspecting the pulmonary tissue as in pulmonary scintigraphy, instead of an arterial assessment as done in conventional CT pulmonary angiography. 

Another evolving CT method for evaluation of perfusion is called CT perfusion. The method is based on conventional CT acquisitions, but adds the time course for the enhancement by serially imaging a tissue volume over time after contrast injection. The method has become an established technique for stroke assessment in most centers, but has also been applied to cardiology, and to a lesser extent for abdominal imaging. In this special edition, CT perfusion was used for treatment evaluation after prostate artery embolization in patients with benign prostatic hyperplasia [7].

## 4. Magnetic Resonance

Modern MRI offers multiple sequences to visualize both vascular anatomy and function. Traditionally both gadolinium-enhanced and non-enhanced methods for visualizing blood vessels have been used for depicting vessel anatomy in all anatomical areas.

2D imaging of vessel flow has been available for many years, but more recently 4D flow MRI has become available for clinical use. Using ECG-gated, time-resolved acquisition, a 3D image of blood flow in all three spatial directions can be obtained. The blood flow of all large blood vessels from the brain to the lower extremities can be visualized. Besides, blood-flow estimation, wall-shear stress, pulse-wave velocity, and pressure gradients may also be assessed [5,6].

There are 3 distinct methods for quantification of tissue perfusion currently clinically available, namely, arterial spin labeling (ASL), dynamic susceptibility contrast (DSC) perfusion, and dynamic contrast-enhanced perfusion (DCE). ASL is performed without the use of gadolinium, and instead uses the ability of MRI to label arterial blood proximal to the image slab. This makes ASL suitable for blood flow estimation in children and in patients with impaired renal function, where the use of gadolinium should be restricted. Both DSC and DCE rely on the administration of gadolinium and offer estimation of blood volume, mean transit time, time to peak, and k-trans. These parameters are mainly used in stroke and brain tumor evaluation.

**Figure 1:**
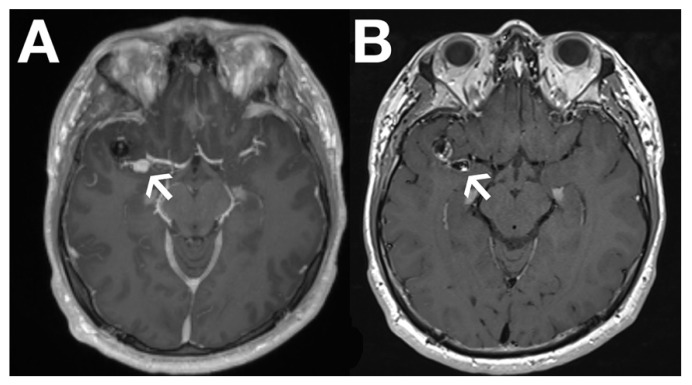
Images show gadolinium-enhanced T1 (**A**) and black blood images (**B**) of a dissection aneurysm following endovascular treatment with a flow diverter of a primary right medial cerebral artery aneurysm. The arrow shows contrast enhancement of the aneurysmal vessel wall on black blood imaging in (**B**), which cannot be seen on the conventional gadolinium-enhanced T1 image in (**A**). The finding indicates an unstable aneurysm.

Recently, black blood imaging has made the evaluation of vessel wall enhancement feasible by suppressing the signal from the blood within the vessel itself. This has proven particularly valuable in brain aneurysm (Figure 1) and in cerebral vasculitis assessment.

## 5. Conclusions

Vascular imaging is expanding with refinements for current techniques and new methods introduced. The focus on and development in vascular imaging are driven by the acknowledgment that flow is fundamental to life and death. The more precise we measure flow, and the more detailed we visualize the vascular system, the better we will understand the dynamics of normal and pathologic processes.
